# Continuous Blood Pressure Estimation Based on Multi-Scale Feature Extraction by the Neural Network With Multi-Task Learning

**DOI:** 10.3389/fnins.2022.883693

**Published:** 2022-05-06

**Authors:** Hengbing Jiang, Lili Zou, Dequn Huang, Qianjin Feng

**Affiliations:** ^1^School of Biomedical Engineering, Southern Medical University, Guangzhou, China; ^2^Institute of Biological and Medical Engineering, Guangdong Academy of Sciences & National Engineering Research Center for Healthcare Devices, Guangzhou, China; ^3^Guangdong Engineering Technology Research Center for Diagnosis and Rehabilitation of Dementia, Guangzhou, China

**Keywords:** continuous blood pressure estimation, multi-scale features, neural networks, multi-task learning, photoplethysmography and electrocardiograph

## Abstract

In this article, a novel method for continuous blood pressure (BP) estimation based on multi-scale feature extraction by the neural network with multi-task learning (MST-net) has been proposed and evaluated. First, we preprocess the target (Electrocardiograph; Photoplethysmography) and label signals (arterial blood pressure), especially using peak-to-peak time limits of signals to eliminate the interference of the false peak. Then, we design a MST-net to extract multi-scale features related to BP, fully excavate and learn the relationship between multi-scale features and BP, and then estimate three BP values simultaneously. Finally, the performance of the developed neural network is verified by using a public multi-parameter intelligent monitoring waveform database. The results show that the mean absolute error ± standard deviation for systolic blood pressure (SBP), diastolic blood pressure (DBP), and mean arterial pressure (MAP) with the proposed method against reference are 4.04 ± 5.81, 2.29 ± 3.55, and 2.46 ± 3.58 mmHg, respectively; the correlation coefficients of SBP, DBP, and MAP are 0.96, 0.92, and 0.94, respectively, which meet the Association for the Advancement of Medical Instrumentation standard and reach A level of the British Hypertension Society standard. This study provides insights into the improvement of accuracy and efficiency of a continuous BP estimation method with a simple structure and without calibration. The proposed algorithm for BP estimation could potentially enable continuous BP monitoring by mobile health devices.

## Introduction

The World Health Organization estimated that nearly 17.9 million people worldwide died of cardiovascular diseases in 2016 (World Health Organization, 2020), posing a serious threat to human health (El-Hajj and Kyriacou, 2020). Blood pressure (BP) monitoring plays an important part in the prevention, diagnosis, and prognosis of cardiovascular disease. The mercury sphygmomanometer is the most common method of measuring BP, but its measured value is instantaneous, random, and might be easily affected by human and environmental factors (O’brien et al., 2003). Therefore, efficient methods are needed to monitor BP continuously and accurately.

Intra-arterial monitoring is the gold standard method for continuous and accurate BP monitoring, but it can result in trauma to the human body and is not suitable for home monitoring. Compared with invasive intra-arterial continuous BP monitoring, non-invasive continuous BP monitoring is more secure and can be measured over a long time. At present, cuff-less arterial tonometry (Pressman and Newgard, 1963) and the volume-compensation method (Penaz, 1973) are mainly used to non-invasive monitor BP continuously. However, arterial tonometry is difficult to operate, which requires professional operation and may be greatly affected by human factors; the volume-compensation method has low precision and needs large equipment. In a word, monitoring theories of these methods limit their wide application in clinical and home use. Therefore, it is necessary to develop an easy-to-use and accurate method for continuous BP monitoring (Lázaro et al., 2019; Yang et al., 2021; Yen and Liao, 2022).

Exploring the relationship between the characteristic parameters of pulse waves and BP is a promising easy-to-use method for continuous and accurate BP monitoring. Recently, many studies have assessed the relationship between pulse wave transit time (PTT) and BP based on traditional methods to estimate BP, but its accuracy is low and these methods need to be calibrated (Chung et al., 2013; Mukkamala et al., 2015; Ding et al., 2016; Huynh et al., 2018). The combination of multiple features (e.g., PTT and pulse wave waveforms features) from photoplethysmography (PPG) and electrocardiograph (ECG) can improve the accuracy of BP estimation (Kachuee et al., 2017; Yoon et al., 2018; Thambiraj et al., 2020). However, these multiple features related to BP from pulse waves are mainly extracted through the feature engineering method, which has been identified as a heavy workload at work, and is difficult to find all of the features from PPG and ECG accurately. Besides, due to the many factors affecting BP, these traditional methods such as support vector machine, random forest, and adaptive boosting, are difficult to accurately fit the relationship between features and BP, which have limited accuracy.

With the development of artificial intelligence especially the deep learning (Ravì et al., 2017; Miotto et al., 2018; Argha et al., 2022), it is possible to extract multiple features related to BP from PPG and ECG and assess their relationship with BP accurately *via* the deep convolutional neural network (Radha et al., 2019; Yan et al., 2019; Li et al., 2020; Song et al., 2020; Paviglianiti et al., 2021). Some researchers have first extracted features manually and then used deep convolutional neural networks to estimate BP (Xu et al., 2017; Yan et al., 2019; Song et al., 2020; Paviglianiti et al., 2021). For example, Xu et al. (2017) have first manually extracted 15 features related to BP from PPG and ECG and then assessed their relationship with BP accurately by using artificial neural networks regression; Maqsood et al. (2021) have first manually extracted time-domain features, statistical features, and frequency domain features and then regressed BP values by using Long Short-Term Memory (LSTM) and Gated Recurrent Unit (GRU) regression. However, these methods also have the disadvantages of inaccuracy and time-consuming of manual feature extraction. Subsequently, the deep neural network methods based on end-to-end learning are used to automatically extract features related to BP and evaluate their relationship with BP, which achieves good performance (Eom et al., 2020). However, to improve the accuracy of BP estimation, the network structure (e.g., the number of network layers) used in these methods are complicated, which would increase the difficulty of model calculation and device deployment. In addition, most of them can only complete one task at a time using their model or complete multi-task using trained multiple models for BP estimation, which greatly reduces the efficiency of BP estimation (Gaurav et al., 2016; Rong and Li, 2021). Therefore, it is necessary to provide a simplified network with high accuracy and efficiency to monitor BP continuously.

In this study, in order to continuously and accurately estimate continuous BP without calibration from ECG and PPG signals, a new method for continuous BP estimation based on multi-scale feature extraction by the neural network with multi-task learning (MST-net) has been proposed and evaluated. Firstly, target signals and label signals arterial blood pressure (ABP) are preprocessed *via* segmenting, extracting labels, denoising, and normalizing. In particular, the interference of abnormal values and the false peak of wave signals are eliminated by peak amplitude and peak-to-peak timing limit. Subsequently, the multi-scale features related to BP are extracted from preprocessed target signals, and the relationship between multi-scale features and BP is trained and learned by the neural network with multi-task learning. Finally, the performance of the neural network is verified and compared with the Association for the Advancement of Medical Instrumentation (AAMI) standards, the British Hypertension Society (BHS) standards, and previous works. This model can not only estimate systolic blood pressure (SBP), diastolic blood pressure (DBP), and mean arterial pressure (MAP) simultaneously but also extract more scale features.

## Materials and Methods

**FIGURE 1:**
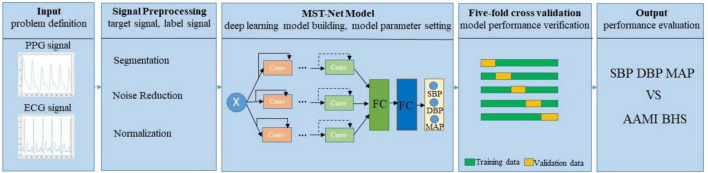
Block diagram of the proposed continuous BP estimation method.

The core concepts of continuous BP estimation based on multi-scale feature extraction by the neural network with multi-task learning proposed in this study are as follows: we adopt segmentation, denoising, and normalization to preprocess the target and label signals, especially using peak-to-peak timing limits of signals to eliminate the interference of the false peak of wave signal; we design a neural network with multi-task learning to extract multi-scale features related to BP from preprocessed target signals, fully excavate and learn the relationship between the multi-scale features and BP, and then estimate three BP values simultaneously through multi-task learning, thus improving the accuracy of BP estimation (Figure 1).

### Problem Definition

To estimate BP continuously and accurately, target signals (PPG and ECG) and label signals (ABP) are synchronously divided into short segments with the same length, which are used as model inputs *x*_*i*_ and reference label BP values (SBP, DBP, and MAP) $y_{i}^{\text{SBP}}$, $y_{i}^{\text{DBP}}$, and $y_{i}^{\text{MAP}}$, respectively. Then all the *x*_*i*_ are used as the input of the neural network, which is used to simultaneously estimate three BP values $z_{i}^{\text{SBP}}$, $z_{i}^{\text{DBP}}$, and ${\text{z}}_{i}^{\text{MAP}}$, and defined as follows:


(1) $$z_{i}^{\text{SBP}}=F\,\left(x_{i};\theta^{\text{SBP}}\right)$$

(2) $$z_{i}^{\text{DBP}}=F\,\left(x_{i};\theta^{\text{DBP}}\right)$$

(3) $$z_{i}^{\text{MAP}}=F\,\left(x_{i};\theta^{\text{MAP}}\right)$$

Where *x*_*i*_ represents input signals, *F*(⋅) represents the input-output function of the neural network with multi-task learning; SBP is the force exerted by blood on arterial walls during ventricular contraction, DBP is the force exerted by the artery walls during ventricular relaxation, and the MAP is the average pressure throughout the cardiac cycle; θ^SBP^, θ^DBP^, and θ^MAP^ represent the specific task parameters of the function; $z_{i}^{\text{SBP}}$, $z_{i}^{\text{DBP}}$, and ${\text{z}}_{i}^{\text{MAP}}$ are the estimated SBP, DBP, and MAP output values of the network, and these three values are produced at the same time through an output layer (3 neurons) followed by the last fully connected (FC) layer.

The convergence of the neural network is evaluated by loss function MSE, and the MSE depends on the difference between the reference label BP values and estimated BP values and is defined as follows:


(4) $$\text{MSE}=\frac{1}{n}\sum_{i=1}^{n}[{\left(y_{\text{i}}^{\text{SBP}}-z_{i}^{\text{SBP}}\right)}^{2}+{\left(y_{\text{i}}^{\text{DBP}}-z_{i}^{\text{DBP}}\right)}^{2}+{\left(y_{\text{i}}^{\text{MAP}}-z_{i}^{\text{MAP}}\right)}^{2}]$$

Where *y*_*i*_ ranges from 60 to 180 mmHg ($60\leq y_{i}^{\text{SBP}},y_{i}^{\text{DBP}},y_{i}^{\text{MAP}}\leq 180$), and *n* is the number of signal segments. The smaller the MSE, the better performance of the model.

### Preprocessing of Signals

Segmentation and label extraction. Our raw data comes from the University of California, Irvine (UCI) Machine Learning Repository dataset (Goldberger et al., 2000; Kachuee et al., 2015), which is derived from the public Multi-parameter Intelligent Monitoring in Intensive Care (MIMIC-II) database. This database contains multiple physiological signals collected simultaneously from intensive care unit patients. In this research, we extract simultaneous recordings of ECG, PPG, and ABP signals of 3,000 subjects from the database which was available at a 125 Hz sampling rate, and select signals with appropriate time (more than 8 min) as the data source. All selected signals were segmented into short segments of 8 s. Subsequently, the peak amplitude limit (80 mmHg ≤ SBP ≤ 180 mmHg, 60 mmHg ≤ DBP ≤ 130 mmHg; Kachuee et al., 2017) and the peak-to-peak time limit (greater than 0.6 s) were set for ABP signal to exclude abnormal value and false peaks. Then, the peaks and troughs were extracted from the processed ABP as reference values of SBP and DBP, respectively. The reference MAP value was calculated as the following formula:


(5) $$\text{MAP}=\frac{\left(\text{SBP}+2\,\text{DBP}\right)}{3}$$

#### Noise Reduction

The segmented ECG and PPG signals are first preprocessed using the discrete wavelet decomposition (DWT) filter with Daubechies 8 wavelet (db8) to remove high-frequency noise, baseline drift, and other noise (Singh and Tiwari, 2006). Specifically, combined with the DWT filter and Nyquist sampling theorem (Unser, 2000), all the ECG and PPG signals are sampled at 125 Hz, and then decomposed layers to extract the approximate coefficients (CAs) and detail coefficients (CDs), respectively. For ECG signals, the number of the decomposed layers is seven, and CDs of the first layer and CAs of the seventh are set to zero to remove the baseline drift (0∼0.5 Hz) and high-frequency noises (31.125∼62.25 Hz); For PPG signals, the number of the decomposed layers is eight, and CDs of the first layer and CAs of the eighth are set to zero to remove the baseline drift (0∼0.25 Hz) and high-frequency noises (31.125∼62.25 Hz). Subsequently, the rest of the CAs and CDs are denoised *via* a soft threshold and then reconstructed to obtain the target PPG and ECG signals.

#### Layer Normalization

Layer normalization is the normalization of a single training data to all neurons in a layer. Through layer normalization, the amplitude of the preprocessed target PPG and ECG signal is distributed within the range of [-1, 1], so that the input signals distribution of the model is similar, and the MST-net model has better converged. The normalization formula is defined as:


(6) $$\text{Normalized}=2\times \frac{x-x_{\min}}{x_{\max}-x_{\min}}-1$$

**FIGURE 2:**
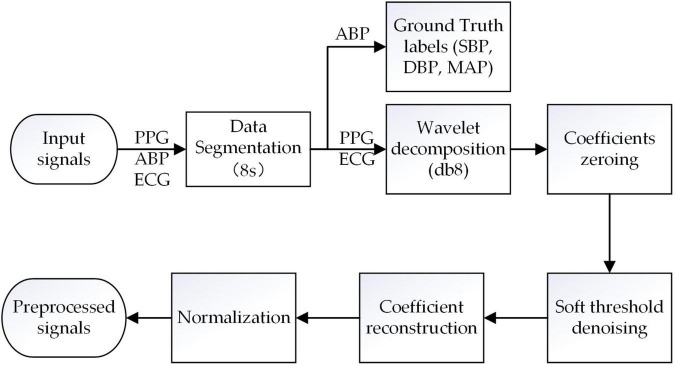
Raw signals preprocessing pipeline.

Where *x* is the amplitude of the target PPG and ECG signals, *x*_*max*_ and *x*_*min*_ are the maximum amplitude and minimum amplitude, respectively, in the target signals. The pre-processing of signals is shown in Figure 2.

### Model Architecture

The core concept of the neural network with multi-task learning we design is as follows (Table 1): we input the ECG and PPG signals (two one-dimensional ECG and PPG; input size: 2 × 1,000) at the same time and then process them using a one-dimensional (1D) convolution layer (Conv; with a convolution kernel of 15) to keep the information of the original signals as much as possible; we utilize the maximum pooling layer to remove redundant information and retain the main signal features; we adopt three network channels whose sizes of convolution kernels are 5, 7, and 9, respectively, to capture multi-scale features related to BP from target signals by using different receptive fields on each channel; we set four modules in each channel and set two convolution layers in each module to extract features; we set 64, 128, 256, and 512 filters on the four modules of each channel, respectively, to learn 1,536 features of target PPG and ECG signals; we set up two FC layers (1,536 and 256 neurons) for regression estimation of BP values, and the output layer consists of three neurons. In addition, to estimate SBP, DBP, and MAP simultaneously, we design a multi-task learning module in the designed neural network to reduce the over-fitting of specific tasks and improve the adaptability and efficiency of different tasks (Ruder, 2017).

**TABLE 1:** The network architecture of the MST-net model.

	MST (5)	MST (7)	MST (9)
		Input (2 × 1,000)	
	Stream 1	Stream 2	Stream 3
Layer 1		Conv (15)	
Layer 2		Max-pooling (3)	
Layer 3	Conv (5)-64	Conv (7)-64	Conv (9)-64
	**	**	**
Layer 4	Conv (5)-64	Conv (7)-64	Conv (9)-64
	**	**	**
Layer 5	Conv (5)-128	Conv (7)-128	Conv (9)-128
	**	**	**
Layer 6	Conv (5)-128	Conv (7)-128	Conv (9)-128
	**	**	**
Layer 7	Conv (5)-256	Conv (7)-256	Conv (9)-256
	**	**	**
Layer 8	Conv (5)-256	Conv (7)-256	Conv (9)-256
	**	**	**
Layer 9	Conv (5)-512	Conv (7)-512	Conv (9)-512
	**	**	**
Layer 10	Conv (5)-512	Conv (7)-512	Conv (9)-512
	**	**	**
Layer 11	AvgPool1d (1)	AvgPool1d (1)	AvgPool1d (1)
Layer 12	FC-512	FC-512	FC-512
Layer 13		FC-256	
		Output-3	

**Represents “Batch Normalization layer + non-linear function rectifier linear unit.”

### Setting of Model Parameter

Batch normalization layer and activation function. Batch normalization is the normalization of individual neurons between different training data. The batch normalization layer can accelerate the convergence rate. Non-linear function rectifier linear unit (RELU) is introduced as the activation function after the normalization layer to avoid the gradient disappearance problem during the training process of the designed network and make the network train faster (Han and Moraga, 1995; Nair and Hinton, 2010; Chung et al., 2015). The RELU formula is defined as follows:


(7) $$\text{Relu}\,\left(x\right)=\left\{ \begin{array}{c} 0,x<0\\ x,x\geq 0 \end{array}\right.$$

#### Adam

Adam can combine the advantages of AdaGrad (adjusting the learning rate (LR) of each different parameter) and Rmsprop (overcome the problem that the gradient of AdaGrad decreases sharply) optimization algorithms to update the parameter of the designed network to find the appropriate parameters and better convergence (Kingma and Ba, 2014).

#### Hyperparameters

In the training process, to train the designed network better and obtain the expected learning effect, the data input batch size is set to 100. The epoch is set to 150. The initial LR of the network is set to 0.01, and the fixed LR is decayed once every 5 epochs, which is defined as follows:


(8) $$\text{LR}=l\,r_{\text{base}}\times {\text{gamma}}^{\frac{\text{step}}{5}}$$

Where *lr*_*base*_ is the original LR, gamma is the decay rate, and step represents the running number of epochs.

#### L2 Regularization

L2 regularization is added in the training process to improve the fitting effects and the generalization performance when the training set is small and the model is complex in the process of designed network training, that is, a constraint term is added to the MSE loss function *L*(θ) (Eq. 3) to generate a new loss function which is defined as follows:


(9) $$L=L\,\left(\theta \right)+\lambda \,\sum_{i=1}^{k}w_{i}^{2}$$

Where *i* is the layer number of the network, *w* is the weight of the network. λ is the coefficient of the L2 regularization which weighs the weight between the constraint term $\sum_{i=1}^{k}w_{i}^{2}$ and *L*(θ). Through the L2 regularization term, *w* can be reduced, and the smaller *w*, the lower the complexity and better the fitting of the network.

### Model Performance Verification

The neural network with multi-task learning proposed in this study runs under the Pytorch1.8.1 framework, using Windows Server 2019 as the operating system. The server is equipped with an RTX 2080ti GPU with 11 G memory and an Intel Xeon Gold 5218 CPU with 32 cores and 64 GB memory. Based on existing methods for creating training/test data sets in BP estimation studies (Yan et al., 2019; Li et al., 2020; Miao et al., 2020a; Panwar et al., 2020), we set different training and test data. Due to the limited number of target signals data sets, five-fold cross-validation is used to evaluate the performance of the model. Our data is randomly divided into five equal-sized subsets, four of which are trained as training data, and the other one is tested as test data in turn, and the average of the five results is used as an estimate of the accuracy of the algorithm.

### Model Performance Evaluation

To evaluate the BP estimation accuracy of the designed neural network, Pearson correlation coefficient (*r*), mean absolute error (MAE), mean error (ME), and standard deviation (SD) are examined. *r* represents the consistency of the estimated BP value and the reference BP value, ME represents the error between the estimated BP value and the reference BP value, MAE represents the absolute error between the estimated BP value and the reference BP value, and SD represents the degree of dispersion between the estimated BP value and the reference BP value. *r*, MAE, ME, and SD equations are defined as follows:


(10) $$r=\frac{\sum_{i=1}^{n}\left(z_{i}-\bar{z}\right)\,\left(y_{i}-\bar{y}\right)}{\sqrt{\sum_{i=1}^{n}{\left(y_{i}-\bar{y}\right)}^{2}\,\sqrt{\sum_{i=1}^{n}{\left(z_{i}-\bar{z}\right)}^{2}}}}$$

(11) $$\text{ME}=\frac{\sum_{i=1}^{n}\left(y_{i}-z_{i}\right)}{n}$$

(12) $$\text{MAE}=\frac{\sum_{i=1}^{n}\left|z_{i}-y_{i}\right|}{n}$$

(13) $$\text{SD}=\sqrt{\frac{\sum_{i=1}^{n}{\left(z_{i}-y_{i}-\text{ME}\right)}^{2}}{n-1}}$$

Where *y*_*i*_ is the reference BP label value obtained from ABP, $\bar{y}$ is the average of *y*_*i*_, *z*_*i*_ is the estimated BP value of the MST-net model, $\bar{z}$ is the average value of *z*_*i*_, and *n* is the total number of target PPG and ECG signals segments in the test data set. Finally, BP estimation accuracy of the MST-net model is compared with the AAMI standards (Association for the Advancement of Medical Instrumentation, 2002) and the BHS standards (O’Brien et al., 1990) which are widely used as criteria for evaluating BP devices.

## Results and Discussion

### Data Source

**FIGURE 3:**
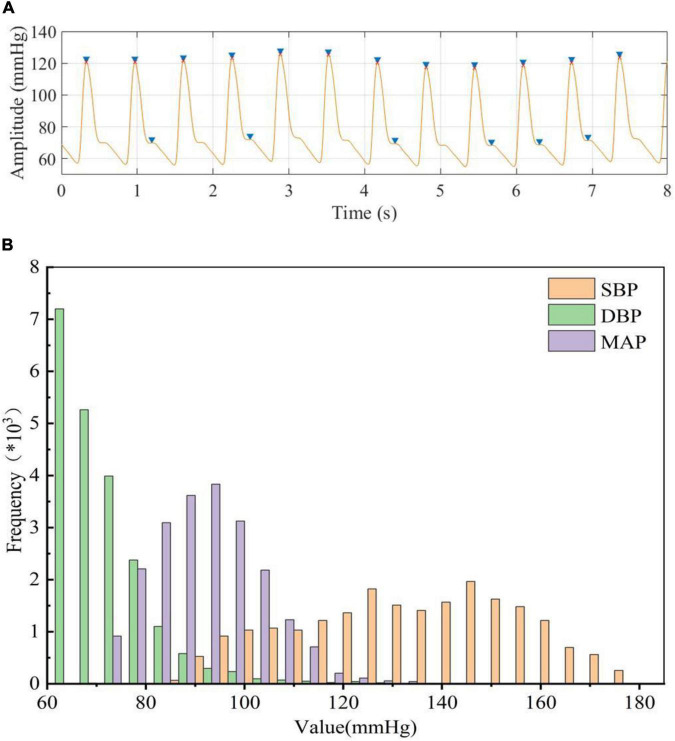
**(A)** blue “▶” represents peak detection before processing, red “x” represents peak detection after processing, **(B)** Statistical histogram of BP data extracted from ABP.

To accurately extract the true BP value (reference BP value) from ABP, the amplitude and peak-to-peak time of the ABP signal were restricted to exclude the interference of abnormal signals and false peaks. We can notice that multiple dicrotic wave peaks (false peaks) existed in the ABP signal (Figure 3A), and this might cause the number of detecting peak values to be more than the true number. We can extract the peak value more accurately after our restriction processing (Figure 3A; red dots). When limited the signal amplitude (<180 mmHg) and time constraints (>8 min) during ABP signals processing, the total number of subjects was reduced from 3,000 to 514. Then we obtained a total of 21,334 segments of BP data after the data segmentation process, that is, we use 213,340 beats in our model. The peaks and troughs were extracted from the processed ABP and used as the reference values of SBP and DBP, respectively, and calculated MAP based on SBP and DBP. The results showed that the DBP was mainly distributed in the range of 60 to 130 mmHg, the SBP was mainly distributed in the range of 80 to 180 mmHg, and the MAP was calculated based on DBP and SBP was mainly distributed between 70 and 135 mmHg (Figure 3B). This result was the same as the distribution of BP values obtained by Miao et al from ABP before (Kachuee et al., 2017; Miao et al., 2020b), which could provide the reference BP values for our designed neural network to estimate BP values.

### Estimation Performance

To judge whether there is overfitting in our model, we used training loss and validation loss during the training process, and the training loss and validation loss were in a stable state after 100 epochs in the model training process, that is, the model had converged.

**FIGURE 4:**
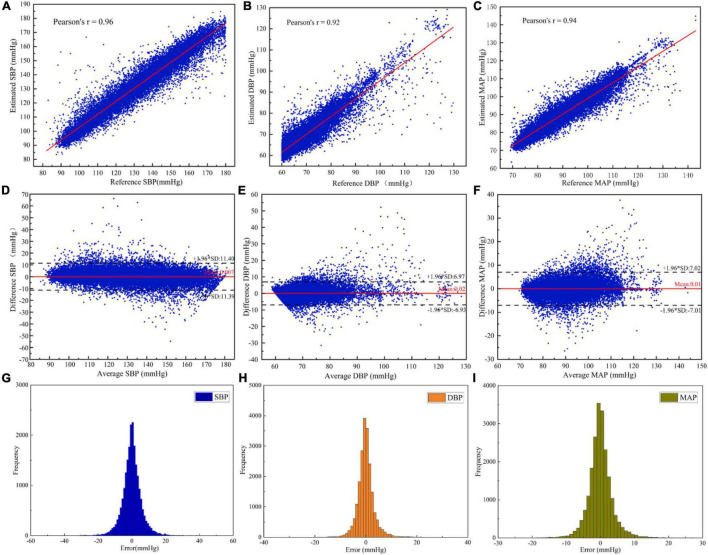
Evaluation of the estimated BP performance of the MST-net model: **(A)** SBP correlation coefficient plot; **(B)** DBP correlation coefficient plot; **(C)** MAP correlation coefficient plot; **(D)** Bland-Altman plot of SBP; **(E)** Bland-Altman plot of DBP; **(F)** Bland-Altman plot of MAP; **(G)** Error histogram for SBP; **(H)** Error histogram for DBP; and **(I)** Error histogram for MAP.

In order to investigate the performance of the designed neural network, the BP estimation accuracy of the network was evaluated according to *r*, ME, MAE, and SD. As result, the r of SBP, DBP, and MAP estimated by the MST-net model were 0.96, 0.92, and 0.94, respectively, and ME ± SD were 0.01 ± 5.81, 0.02 ± 3.55, and 0.01 ± 3.58 mmHg, respectively, (Figure 4). It can be observed that all reference values have a strong linear relationship with BP estimates (SBP, DBP, and MAP). The *p*-value for SBP, DBP, and MAP were 0.972, 0.796, and 0.969, respectively. It implied that the population mean of the samples was equal. These data points fell on both sides of the regression line and were close to the regression line (Figures 4A–C), indicating estimated BP data with high accuracy. The average values and difference values of reference and estimated BP values were the horizontal axis and vertical axis of the Bland-Altman plot (Figures 4D–F), respectively, these data points fall within the 95% confidence interval [-1.96 × SD, 1.96 × SD], indicating a good level of consistency of the reference and estimated BP data. Also, the average error between the reference and the estimate (red line) is very close to zero mmHg, indicating a high degree of consistency between the reference and estimated BP data. In addition, we provided the histogram of the error distribution between the estimate and the reference value, and we can observe that most of the errors are concentrated around 0 (Figures 4G–I). On the other hand, our network had fewer parameters than previous network models (Biswas et al., 2019; Panwar et al., 2020). That is to say, our network with an optimized algorithm was of lower complexity which can contribute to avoiding the constraints of computing power and memory for platform deployments (e.g., mobile devices, wearable devices). From the above analysis, it can be noted that the method of this study can achieve a precision estimation of SBP, DBP, and MAP. It is noted that the estimated SBP values through our model were not limited to less than 180 mmHg, and the SBP values beyond 180 mmHg can be also predicted through our model. However, we just predicted SBP values within 180 mmHg same as many references (Kachuee et al., 2017; Baek et al., 2019; Thambiraj et al., 2020, etc.). The reasons were as follows: First, there were a few cases of SBP reaching 180 mmHg in the database; Secondly, when analyzing the reference signal and input signal values, the SBP values greater than 180 mmHg were calculated from the reference signal, which was generally caused by irregular noise signals.

In order to evaluate the accuracy of BP estimation based on this study, the BP estimation results of this study were compared with international AAMI and BHS standards. According to the AAMI standard (Table 2), the target subjects of this study were 514, far more than the 85 required by the AAMI standard. the ME of SBP, DBP, and MAP were 0.007, 0.022, and 0.009 mmHg, respectively, which were far lower than the ME ≤ 5 mmHg required by the AAMI standard. SD of SBP, DBP, and MAP estimated were 5.81, 3.55, and 3.58 mmHg, respectively, which were far lower than SD ≤ 8 mmHg required by the AAMI standard. It showed that the estimated BP values by our customized model met the AAMI standard. The cumulative error percentage estimated by our model of the SBP reached 71.56, 92.28, and 97.66%, respectively; the DBP reached 89.88, 98.25, and 99.40%, respectively, and the MAP reached 87.89, 98.03, and 99.52%, respectively, (Table 3), which all showed much higher than the A grade standard of BHS (60, 85, and 95%). To sum up, BP values (SBP, DBP, and MAP) estimated by our customized model reached a small error and achieved good results.

**TABLE 2:** Comparison of estimated BP values between our work and AAMI standard.

		ME (mmHg)	SD (mmHg)	Subjects	Assessment result
Our results	SBP	0.007	5.81		
	DBP	0.022	3.55	514	Satisfied
	MAP	0.009	3.58		
AAMI	SBP	≤5	≤8	≥85	
(AAMI, 2002)	DBP				
	MAP				

**TABLE 3:** Comparison of estimated BP values between our work and BHS standard.

		Cumulative error percentage			
		C. P. 5	C. P. 10	C. P. 15	Assessment
					result
	SBP	71.56%	92.28%	97.66%	A
Our result	DBP	89.88%	98.25%	99.40%	A
	MAP	87.89%	98.05%	99.52%	A
	Grade A	60%	85%	95%	
BHS (O’Brien et al., 1990)	Grade B	50%	75%	90%	
	Grade C	40%	65%	80%	

In order to verify the effectiveness of the designed network, the proposed BP estimation method was compared with previous work. In general, it is difficult to make a fair comparison of BP estimation work due to different evaluation metrics and inadequately specified datasets. For example, for some BP estimation work based on ECG and PPG, Miao et al. (2020a) and Ding et al. (2016) used their own data sets, Baker et al. (2021) used the MIMIC III database, Sharifi et al. (2019) used the same database like ours. From the results, our proposed method performed better than these studies (Table 4). For a fair comparison, we selected two popular machine learning methods, VGG network (Simonyan and Zisserman, 2015) and Resnet (He et al., 2016), to compare BP estimation results using the same dataset. We can notice that the MAE ± SD of SBP, DBP, and MAP in our study (SBP: 4.04 ± 5.81, DBP: 2.29 ± 3.55, and MAP: 2.46 ± 3.39) all were smaller than the popular machine learning methods results (VGG, SBP: 8.47 ± 11.45, DBP: 4.70 ± 6.70, MAP: 5.09 ± 6.94; Resnet, SBP: 4.12 ± 5.97, DBP: 2.31 ± 3.60, and MAP: 2.50 ± 3.67), indicating that the network model we designed performed better at a BP estimated work (Table 5). It should be noted that the setting of hyperparameters in the two popular machine learning methods is the same as our proposed method (except that the LR of the VGG network is set to 0.005). In addition, we noticed that compared with previous studies that used a separate training method to estimate a BP value, our model can simultaneously estimate multiple BP values by using only one model, which not only reduced the complexity but also improved the work efficiency (Table 6).

**TABLE 4:** Comparison with other experimental performance.

Work	Dataset	Method	SBP		DBP		MAP	
			MAE	SD	MAE	SD	MAE	SD
Miao et al., 2020a	Own dataset	Feature extraction	6.13	7.76	4.54	5.52	4.81	6.03
Ding et al., 2016			4.09	5.21	3.18	4.13	3.18	4.06
Sharifi et al., 2019	MIMIC II	Deep learning algorithm	7.83	9.10	4.86	5.21	3.63	4.60
Baker et al., 2021	MIMIC III		4.41	6.11	2.91	4.23	2.77	3.88
This work	MIMIC II	MST deep learning algorithm	4.04	5.81	2.29	3.55	2.46	3.39

**TABLE 5:** Comparison of predicted BP values between our and previous work based on our dataset from MIMIC-II.

Work	MAE ± SD (mmHg)		
	SBP	DBP	MAP
Resnet (He et al., 2016)	4.12 ± 5.97	2.31 ± 3.60	2.50 ± 3.67
VGG (Simonyan and Zisserman, 2015)	8.47 ± 11.45	4.70 ± 6.70	5.09 ± 6.94
This work	4.04 ± 5.81	2.29 ± 3.55	2.46 ± 3.39

**TABLE 6:** Comparison of No. model for BP evaluation between our and previous work.

Work	Subjects	Model	SBP (mmHg)	DBP (mmHg)
Rong and Li, 2021	11,546 samples	2	5.59 ± 7.25	3.36 ± 4.48
Kachuee et al., 2017	942 subjects	2	11.17 ± 10.09	5.35 ± 6.14
Gaurav et al., 2016	3,000* subjects	2	4.47 ± 6.85	3.21 ± 4.72
This work	21,334 samples	1	4.04 ± 5.81	2.29 ± 3.55

*Number of subjects before signal processing.

Overall, the MST-net model proposed in this study had a relatively simple structure and achieved good accuracy in the field of continuous BP estimation, which was a very competitive method and made contributions to the improvement of BP estimation accuracy.

### Impacts of Model Architecture

In order to determine the effects of network structure on the performance of BP estimation, the effects of the number of network channels and the size of the convolution kernel of network channels on BP estimation performance were investigated (Table 7). Compared to the BP estimation results of the single-channel model with convolution kernels of 7, the estimation errors of SBP, DBP, and MAP *via* the three-channel model with convolution kernels of (3, 5, 7) were reduced by 0.03, 0.05, and 0.05 mmHg, respectively. The reason was that multi-channel can extract more abundant features than single-channel. In the single-channel model, when the size of the convolution kernel increased from 3 to 9, the errors of SBP, DBP, and MAP decreased significantly, indicating that the increase of the convolution kernel in the model could improve BP estimation performance. The reason was that the larger convolution kernel has a larger receptive field which contributes to extracting the features related to BP from time-series signals with periodic patterns. These results also showed that the features related to BP have a larger span of time. At the same time, with the increase in the size of the convolution kernel, the increase of BP estimation performance gradually decreased, indicating that the size of the convolution kernel used to extract features was limited, and cannot be infinite. When the size of the multi-channel convolution kernel was increased from (3, 5, 7) to (5, 7, 9), the estimated errors of SBP, DBP, and MAP were improved by 0.06, 0.01, and 0.01 mmHg, respectively. This also showed that the larger convolution kernel could improve the performance of the BP estimation model.

**TABLE 7:** Impacts of that number of network channels and the size of channel convolution kernel on the performance of BP estimation.

Kernel size	SBP (mmHg)	DBP (mmHg)	MAP (mmHg)
MST-net (3)	4.40	2.50	2.68
MST-net (5)	4.20	2.41	2.58
MST-net (7)	4.13	2.33	2.52
MST-net (9)	4.07	2.31	2.48
MST-net (3, 5, 7)	4.10	2.28	2.47
MST-net (5, 7, 9)	4.04	2.29	2.46

## Conclusion

In this article, a novel continuous BP estimation based on multi-scale feature extraction by the neural network with multi-task learning was proposed to estimate BP continuously and accurately without calibration using PPG and ECG signals. This research was a step toward developing an efficient and lightweight solution. We adopted segmenting, denoising, and normalizing to preprocessed target and label signals and then extracted the reference BP value from the preprocessed label signals, especially using peak-to-peak timing limits of signals to eliminate the interference of the false peak of the wave; we designed a neural network with multi-task learning to extract multi-scale features related to BP from preprocessed target signals, fully excavated and learned the relationship between the multi-scale features and BP, and then estimated three BP values simultaneously through multi-task learning. The results showed that the errors of MAE ± SD for SBP, DBP, and MAP were 4.04 ± 5.81, 2.29 ± 3.55, and 2.46 ± 3.58 mmHg, respectively, and the correlation coefficients were 0.96, 0.92, and 0.94, respectively. These results met the AAMI standard and reached A level of the BHS standard, and showed better BP continuous monitoring results than other previous works, and without calibration.

In conclusion, our study provided convincing evidence that our method can achieve high precision continuous BP estimation and had a relatively simple structure, which can be further applied to wearable devices.

## Data Availability Statement

The original contributions presented in the study are included in the article/supplementary material; further inquiries can be directed to the corresponding authors.

## Author Contributions

HJ, LZ, and QF: conceptualization. HJ: methodology, software, and data curation. HJ and LZ: formal analysis and original draft preparation. LZ, QF, and DH: review and editing, supervision, and funding acquisition. All authors have read and agreed to the published version of the manuscript.

## Conflict of Interest

The authors declare that the research was conducted in the absence of any commercial or financial relationships that could be construed as a potential conflict of interest.

## Publisher’s Note

All claims expressed in this article are solely those of the authors and do not necessarily represent those of their affiliated organizations, or those of the publisher, the editors and the reviewers. Any product that may be evaluated in this article, or claim that may be made by its manufacturer, is not guaranteed or endorsed by the publisher.
